# Short- and long-term neonatal outcomes according to differential exposure to antenatal corticosteroid therapy in preterm births prior to 24 weeks of gestation

**DOI:** 10.1371/journal.pone.0198471

**Published:** 2018-06-04

**Authors:** Seon-Mi Kim, Ji-Hee Sung, Jin-Yi Kuk, Hyun-Hwa Cha, Suk-Joo Choi, Soo-young Oh, Cheong-Rae Roh

**Affiliations:** 1 Department of Obstetrics and Gynecology, Samsung Medical Center, Sungkyunkwan University School of Medicine, Seoul, Korea; 2 Department of Obstetrics and Gynecology, Kangbuk Samsung Hospital, Sungkyunkwan University School of Medicine, Seoul, Korea; 3 Department of Obstetrics and Gynecology, Kyungpook National University Hospital, Kyungpook National University School of Medicine, Daegu, Korea; University of Illinois at Urbana-Champaign, UNITED STATES

## Abstract

**Aim:**

To assess the effects of differential exposure to antenatal corticosteroid (ACS) on short- and long-term outcomes of infants born before 24 weeks of gestation.

**Methods:**

This is a retrospective cohort study of 147 infants delivered by 116 women at 21–23 weeks of gestation between January 2001 and December 2016 at a tertiary referral hospital in Seoul, Korea. Eligible subjects were categorized into the following three groups according to ACS exposure: non-user (n = 53), partial-course (n = 44), and complete-course (n = 50). Univariable and multivariable analyses were used to compare neonatal mortality, neonatal morbidities including intraventricular hemorrhage (IVH), and neurodevelopmental impairment including cerebral palsy among the three groups.

**Results:**

Neonatal mortality rate was significantly lower in the ACS-user groups (non-user, 52.8%; partial-course, 27.3%; complete-course, 28.0%; P = 0.01), but complete-course of ACS therapy had no advantages over partial-course. A lower incidence of IVH was observed in the complete-course group (non-users, 54.8%; partial-course, 48.6%; complete-course, 20.5%; P = 0.003). Multiple logistic regression analysis showed that ACS therapy, either partial- or complete-course, was associated with a lower rate of neonatal mortality (adjusted odds ratio (aOR) 0.375; 95% confidence interval (CI) 0.141–0.996 in partial-course; aOR 0.173; 95% CI 0.052–0.574) in complete-course). IVH (aOR 0.191; 95% CI 0.071–0.516) was less likely to occur in the complete-course group than in the non-user group. Neurodevelopmental impairment of survivors at 18–22 month after birth was not significantly different among the three groups.

**Conclusion:**

ACS therapy in preterm births at 21–23 weeks of gestation was associated with significantly reduced rates of neonatal mortality and IVH, especially with complete administration.

## Introduction

Preterm births prior to 24 weeks of gestation comprise only <1% of all pregnancies, but the number has increased, raising issues over health care costs and ethical questions [1]. One of the most proven methods to improve outcomes for preterm infants is antenatal corticosteroids (ACS). However, ACS therapy for preterm birth before 24 weeks of gestation is generally not recommended because its clinical efficacy remains unclear.

According to recently updated guidelines of the Royal College of Obstetricians and Gynecologists, the consensus statement of the American College of Obstetricians and Gynecologists, and New Zealand-Australia guidelines, ACS therapy should be considered from 23 weeks of gestation after careful evaluation of the benefits and risks with parenteral consultation [2–4]. These guideline changes were based on recent evidence of the clinical benefit of ACS therapy before 24 weeks of gestation. Recent meta-analyses of randomized controlled trials indicated that the use of ACS in peri-viable infants reduced neonatal deaths or severe neonatal complications [5,6]. However, the numbers of infants born prior to 24 weeks of gestation in these studies were too small to have power to conclude whether ACS therapy is effective for these populations. Without clear evidence, the National Institutes of Child Health and Human Development (NICHD) have not recommended ACS therapy for peri-viable births in 1994, 2001, 2008, and 2011 recommendations [7]. The lack of clinical guidelines contributes to regional and international variation of clinical practice in these patients.

In addition, the effect of ACS therapy is most beneficial when the baby is delivered between 24 hours and 7 days after the administration of complete course [8,9]. However, a large proportion of women at risk of birth prior to 24 weeks of gestation give birth without completing the ACS administration schedule, mostly because of urgent delivery. Therefore, the aim of our study was to assess the short- and long-term outcomes of infants born at 21–23 weeks of gestation and to investigate the effect of differential exposure of ACS (partial vs. complete) on neonatal outcome.

## Methods

We conducted a retrospective cohort study at a tertiary referral hospital in Seoul, Korea, from 2001 to 2016. Pregnant women admitted to our institute from January 2001 to December 2016 were screened by reviewing electronic medical records. Among them, patients who delivered between 21 weeks 0 day and 23 weeks 6 days of gestation were considered eligible. Gestational age was determined by the patient’s last menopausal period or crown-rump length on first trimester ultrasonography. Exclusion criteria included stillbirth, cases of voluntary termination of pregnancy or parental elective non-resuscitations, delayed-interval delivery in twin pregnancies, and major congenital anomaly. Demographic information and obstetrical and neonatal outcomes, along with type of ACS used and antenatal tocolytic treatment regimens were reviewed from maternal and neonatal medical records. The study protocol was approved by the Institutional Review Board of Samsung Medical Center.

First, we analyzed the incidence of preterm birth at 21–23 weeks of gestation, neonatal mortality rate, infant mortality rate, and rate of administration of ACS therapy in preterm births at 21–23 weeks of gestation from 2001 to 2016 in our hospital. Neonatal mortality was defined as death before 28 days of life, and infant mortality as death before 1 year of age. Subjects were categorized into four subgroups according to year of delivery: 2001 to 2004, 2005 to 2008, 2009 to 2012, and 2013 to 2016. The incidence of preterm birth at 21–23 weeks of gestation, neonatal mortality rate, infant mortality rate, and rate of administration of ACS therapy were calculated for each period of time, with respect to weeks of gestation at delivery (21, 22, and 23 weeks of gestation).

Subjects were grouped according to ACS exposure: non-user, partial-course, and complete-course groups. Complete-course ACS therapy was defined as administration of four intramuscular injections of 6 mg dexamethasone per 12 hours or two intramuscular injections of 12 mg betamethasone per 24 hours before delivery. Partial-course ACS therapy was defined as incomplete corticosteroid administration (1 dose of betamethasone or less than 4 doses of dexamethasone) before delivery. At our institute, we recommend pregnant women at risk of preterm birth before 24 weeks of gestation consult the obstetrician and neonatologist regarding the treatment and prognosis of extreme premature infants and make decisions on continuing pregnancy and neonatal resuscitation therapy. When pregnant women choose to continue the pregnancy and resuscitate the neonates, we prescribe ACS therapy. However, in case of emergent deliveries, ACS therapy cannot be carried out or only partially administered.

The primary outcome variables of this study were neonatal mortality and major neonatal morbidities including respiratory distress syndrome (RDS), broncho-pulmonary dysplasia (BPD), intra-ventricular hemorrhage (grade ≥3), peri-ventricular leukomalacia (PVL), patent ductus arteriosus (PDA), grade III or IV retinopathy of preterm (ROP), necrotizing enterocolitis (NEC) worse than grade II, neonatal sepsis, and long-term neurodevelopmental outcome. RDS was defined as clinical symptoms of respiratory distress (tachypnea, substernal or intercostal retraction, nasal flaring, grunting, or cyanosis) requiring oxygen supplement with a fraction of inspired oxygen (FiO2) greater than 0.4 and compatible chest simple radiography findings of diffuse reticular opacification of both lungs (ground-glass appearance) with air bronchograms and hypoaeration. BPD was diagnosed when supplemental oxygen with FiO2 greater than 0.4 was required for the first 28 days of life [10]. IVH was defined and graded by classification according to Papile et al. [11]. PVL was diagnosed if increased echogenicity and cystic lesions were identified in the periventricular white matter under cranial ultrasonography. Grade III or IV IVH was considered an outcome variable due to poor prognosis. PDA was evaluated by echocardiography within 10 days after birth. ROP was clinically diagnosed and graded according to the international classification of ROP, where grade III or IV ROP was considered a clinical variable. Neonates who presented with abdominal distension and hematochezia and showed pneumatosis intestinalis on abdominal radiograph were diagnosed with NEC (graded by modified Bell’s staging criteria) [12]. Neonatal sepsis was diagnosed by clinical findings and presence of bacteria or fungus in the blood culture. Neonates who presented with only clinical symptoms or signs of sepsis were classified as “suspected neonatal sepsis.” Neonatal sepsis was divided into early onset (if symptoms started before 72 hours of life) and late onset (if symptoms started after 72 hours of life) [13,14]. Other neonatal outcomes analyzed were birth weight, small for gestational age, 1- and 5-minute Apgar scores, rate of admission to neonatal intensive care unit (NICU), duration of NICU stay, and duration of ventilator requirement if mechanical ventilation was applied.

The neurodevelopmental outcomes of the surviving infants were analyzed at 18 to 22 months after birth. Neurodevelopmental impairment (NDI) was defined as the presence of any of the following: 1) cerebral palsy (CP) with level III or higher grade according to gross motor function classification system (GMFCS) [15,16], 2) mental developmental index less than 70 on the Bayley scales of infant and toddler development version II (BSID-II) [17] or cognitive score less than 85 on BSID-III [18], 3) suspicion for developmental delay on Denver Developmental Screening test (DDST-II) [19], and 4) blindness or use of cochlear implant due to hearing loss or deafness. The BSID-II or BSID-III system was applied to infants born in 2001–2005 or 2006–2016, respectively. DDST-II data were used in subjects who were not able to carry out the BSID test or if parents did not agree to the BSID test. All of above methods were carried out as per routine clinical care.

The null hypothesis tested of this study was that there are no differences in the maternal and neonatal outcomes according to different exposure to ACS therapy. Chi-square test was used to compare categorical variables of three independent groups. Analysis of variance (ANOVA) was used to compare continuous and normally distributed variables, and Kruskal-Wallis test was used to compare continuous variables with skewed distribution. Two-tailed P-value less than 0.05 was considered statistically significant. For multiple comparisons, post-hoc analysis with Bonferroni correction was applied, and the P-value was adjusted to 0.0083 (0.05/6). The Jonckheere-Terpstra test and linear-by-linear association were used for trend analysis in continuous and categorical variables, respectively. Multivariable logistic regression analysis was performed to calculate adjusted odds ratio (aOR) of neonatal outcome according to ACS use adjusted for maternal age, birth weight, neonatal sex, multiplicity of pregnancy, year of birth, use of tocolytics, admission-to-delivery interval, gestational age at delivery, and mode of delivery.

## Results

During the 16-year period of review, 228 cases of preterm birth before 24 weeks of gestation were identified from 34,183 deliveries in our institute. Among them, 116 women met the selection criteria and were enrolled to the study. Twenty-nine of these women had twin pregnancy. One hundred forty-seven infants born from these 116 women were analyzed for neonatal outcomes.

**Fig 1:**
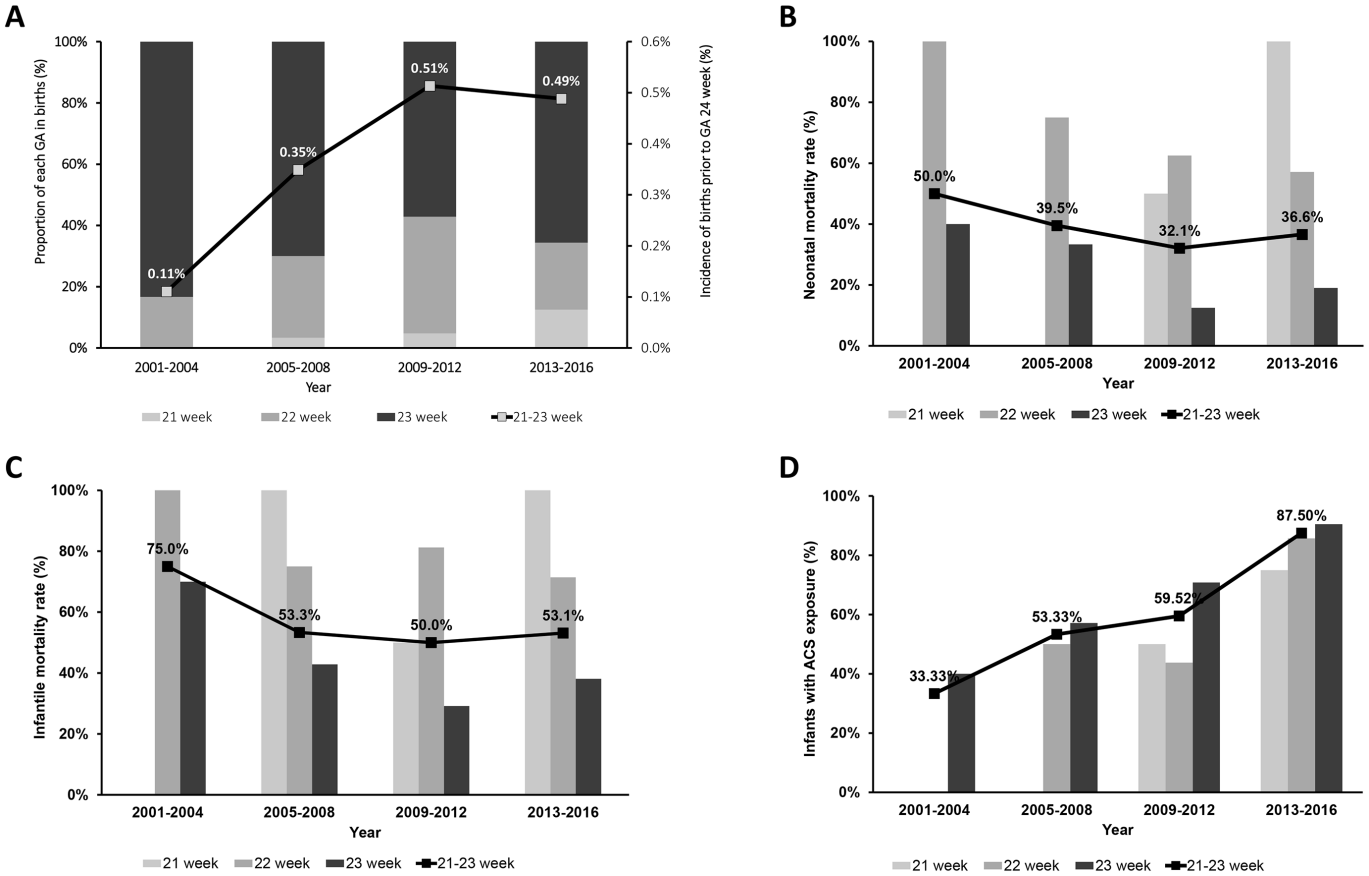
Incidence of preterm birth at 21–23 weeks of gestation, neonatal mortality rate, infant mortality rate, and rate of ACS administration in preterm births at 21–23 weeks of gestation according to the time period; 2001 to 2004, 2005 to 2008, 2009 to 2012, and 2013 to 2016. The incidence of preterm births at 21–23 weeks of gestation increased significantly over time (A). Neonatal mortality rate (B) and infant mortality rate (C) decreased over time, but without statistical significance. A significant increasing trend of ACS therapy was observed (D).

The incidence of preterm births at 21–23 weeks of gestation increased significantly (Fig 1A). Most of these preterm births occurred at 22 or 23 weeks of gestation (94.0%); however, the proportion of preterm birth at 21 weeks of gestation increased significantly over time (2001–2004: 0%; 2005–2008: 2.9%; 2009–2012: 3.9%; 2013–2016: 12.2%, P < 0.001). Neonatal mortality rate (Fig 1B) and infant mortality rate (Fig 1C) declined, but without statistical significance. The rate of ACS administration increased significantly over time (Fig 1D).

Maternal age, obstetric history, twin pregnancy, indication for admission, use of tocolytics and ACS, and presence of histological chorioamnionitis were not significantly different according to gestational age at delivery, but cesarean section rate increased with gestational age at delivery (Table 1). There was a significant trend of decreasing rates of low Apgar scores at 1 and 5 minutes, neonatal mortality, infant mortality, and necrotizing enterocolitis with increasing gestational age at delivery (Table 2). Other outcomes including long-term neurodevelopmental outcome were not significantly different among infants born at 21, 22, or 23 weeks of gestation.

**Table 1:** Maternal characteristics and pregnancy outcomes according to gestational age at birth.

	21 weeks (n = 7)	22 weeks (n = 33)	23 weeks (n = 76)	P-value^a^
Age (year, mean±SD)	35.9±3.1	31.5±4.2	32.7±3.9	0.793
Nulliparity	4 (57.1%)	18 (54.5%)	41 (53.9%)	0.884
History of PTD	1 (14.3%)	5 (15.2%)	14 (18.4%)	0.653
Multiple pregnancy	3 (42.9%)	8 (24.2%)	18 (23.7%)	0.424
Admission indication				
PTL or IIOC	6 (85.7%)	27 (81.8%)	55 (72.4%)	0.131
PPROM	1 (14.3%)	5 (15.2%)	13 (17.1%)	
Maternal/fetal	0 (0%)	1 (3.0%)	8 (10.5%)	
Tocolytics	6 (85.7%)	24 (72.7%)	51 (67.1%)	0.287
Nifedipine	1 (14.3%)	13 (39.4%)	15 (19.7%)	0.249
Ritodrine	6 (85.7%)	21 (63.6%)	47 (61.8%)	0.335
Magnesium sulfate	0 (0%)	2 (6.1%)	12 (15.8%)	0.083
Atosiban	1 (14.3%)	4 (12.1%)	16 (21.1%)	0.317
ACS therapy	4 (57.1%)	17 (51.5%)	52 (68.4%)	0.145
Dexamethasone^b^	0 (0%)	1 (5.9%)	5 (9.6%)	0.440
Betamethasone^b^	4 (100%)	16 (94.1%)	47 (90.4%)	
Partial-course^b^	3 (75.0%)	6 (35.3%)	24 (46.2%)	0.778
Complete-course^b^	1 (25.0%)	11 (64.7%)	28 (53.8%)	
Cesarean delivery	0 (0%)	15 (45.5%)	44 (57.9%)	0.006
Histological chorioamnionitis	5 (71.4%)	27 (81.8%)	53 (69.7%)	0.374

SD; standard deviation, PTD; preterm delivery, PTL; preterm labor, IIOC; incompetent internal os of cervix, PPROM; preterm premature rupture of membrane, ACS; antenatal corticosteroid

^a^ Jonckheere-Terpstra test for continuous variables and linear-by-linear regression for categorical variables

^b^ Proportion of subjects who received ACS therapy

**Table 2:** Neonatal outcomes according to gestational age at birth.

	21 weeks (n = 10)	22 weeks (n = 41)	23 weeks (n = 96)	P-value^a^
Sex (Male)	3 (30.0%)	23 (56.1%)	49 (51.0%)	0.571
Birth weight (g, mean±SD)	466.0±44.5	503.1±75.2	580.4±91.3	0.167
SGA	3 (30.0%)	11 (26.8%)	14 (14.6%)	0.067
1 min Apgar score <4	7 (70.0%)	23 (56.1%)	38 (39.6%)	0.019
5 min Apgar score <7	8 (80.0%)	26 (63.4%)	38 (39.6%)	0.001
NICU admission	10 (100%)	41 (100%)	96 (100%)	-
Duration of NICU stay (days, median [range])^b^	12.5 [1–169]	18 [1–380]	118.5 [1–321]	<0.001
Ventilator treatment	10 (100%)	41 (100%)	96 (100%)	-
Duration of assisted ventilation (days, median [range])^c^	12.5 [1–63]	18 [1–270]	48 [1–212]	0.046
Neonatal mortality	7 (70.0%)	26 (63.4%)	21 (21.9%)	<0.001
Infant mortality	9 (90.0%)	32 (78.0%)	34 (35.4%)	<0.001
Neonatal morbidity^d^				
RDS	10/10 (100%)	41/41 (100%)	96/96 (100%)	-
BPD	3/3 (100%)	15/15 (100%)	74/75 (98.7%)	0.644
IVH (≥grade 3)	2/5 (40.0%)	11/30 (36.7%)	37/88 (42.0%)	0.674
PVL	0/5 (0%)	5/29 (17.2%)	14/88 (15.9%)	0.626
ROP (≥grade 3)	0/1 (0%)	4/12 (33.3%)	28/73 (38.4%)	0.502
NEC (≥stage 2)	3/6 (50.0%)	10/27 (37.0%)	17/83 (20.5%)	0.029
Early sepsis	9/9 (100%)	24/34 (70.6%)	60/87 (69.0%)	0.131
Late sepsis	2/5 (40.0%)	16/28 (57.1%)	55/83 (66.3%)	0.176
Neurodevelopmental outcome^e^				
NDI	-	8/8 (100%)	48/54 (88.9%)	1.000^f^
Developmental delay by BSID or DDST	0/1 (0%)	8/8 (100%)	47/55 (85.5%)	0.581^f^
Cerebral palsy	0/1 (0%)	5/9 (55.6%)	33/59 (55.9%)	1.000^f^
Cerebral palsy ≥ level III	-	1/5 (20.0%)	3/32 (9.4%)	0.456^f^

SD; standard deviation, PTD; preterm delivery, SGA; small-for-gestational age, NICU; neonatal intensive care unit, RDS; respiratory distress syndrome, BPD; bronchopulmonary dysplasia, IVH; intraventricular hemorrhage, PVL; periventricular leukomalacia, ROP; retinopathy of prematurity, NEC; necrotizing enterocolitis, NDI; neurodevelopmental impairment, BSID; Bayley Scales of Infant and Toddler Development, DDST; Denver Developmental Screening test

^a^ Jonckheere-Terpstra test for continuous variables and linear-by-linear regression for categorical variables

^b^ analyzed with infants admitted to the NICU only

^c^ analyzed with infants treated with assisted ventilation only

^d^ denominators are the numbers of cases who survived until the diagnosis of each morbidity was possible

^e^ denominators are the numbers of cases who survived until the BSID or neurological examination was possible

^f^ statistical analysis between 22 and 23 weeks of gestation

Subjects were allocated to three groups according to ACS therapy: 43 women were non-users, 33 women received partial-course, and 40 women received complete-course. Two women received repeated course of ACS after 7 days of the initial complete course and delivered at 2 days after the second course, and they were included in the complete-course group. Maternal age, obstetric history, multiple pregnancy, and indication for admission were similar in the three groups, while gestational age at admission, use of tocolytics, and ACS type were significantly different among the three groups (Table 3). Gestational age at delivery, mode of delivery, and presence of histological chorioamnionitis were not significantly different among the three groups.

**Table 3:** Maternal characteristics and pregnancy outcomes according to exposure to antenatal corticosteroid therapy.

	Non-user (n = 43)	Partial-course (n = 33)	Complete-course (n = 40)	P-value^a^
Age (year, mean±SD)	32.88±4.40	32.06±3.61	32.65±3.63	0.656
Nulliparity	21 (48.8%)	20 (60.6%)	22 (55.0%)	0.590
History of PTD	7 (16.3%)	4 (12.1%)	9 (22.5%)	0.494
Multiple pregnancy	9 (20.9%)	12 (36.4%)	8 (20.0%)	0.203
Admission indication				
PTL or IIOC	33 (76.7%)	26 (78.8%)	29 (72.5%)	0.930
PPROM	6 (14.0%)	5 (15.2%)	8 (20.0%)	
Maternal/fetal	4 (9.3%)	2 (6.1%)	3 (7.5%)	
GA at admission (weeks, mean±SD)	22.6±1.1	23.1±0.7^b^	22.1±1.4	0.002
Tocolytics	19 (44.2%)	29 (87.9%)^b^	33 (82.5%)^b^	<0.001
Nifedipine	7 (16.3%)	5 (15.2%)	17 (42.5%)^b^	0.007
Ritodrine	18 (41.9%)	28 (84.8%)^b^	28 (70.0%)^b^	<0.001
Magnesium sulfate	2 (4.7%)	6 (18.2%)	6 (15.0%)	0.156
Atosiban	2 (4.7%)	6 (18.2%)	13 (32.5%)^b^	0.004
Type of ACS used				
Dexamethasone	-	6 (18.2%)	0 (0%)	0.007
Betamethasone	-	27 (81.8%)	40 (100%)	
GA at delivery (weeks, mean±SD)	22.9±0.7	23.2±0.6	23.2±0.6	0.158
Cesarean delivery	16 (37.2%)	20 (60.6%)	23 (57.5%)	0.076
Histological chorioamnionitis	28 (65.1%)	26 (78.8%)	31 (77.5%)	0.311

SD; standard deviation, PTD; preterm delivery, PTL; preterm labor, IIOC; incompetent internal os of cervix, PPROM; preterm premature rupture of membrane, GDM; gestational diabetes mellitus, GA; gestational age, ACS; antenatal corticosteroid

^a^ intergroup difference by analysis of variance or Kruskal Wallis test continuous variables, and Chi-square test for categorical variables

^b^ significantly different compared to the non-user group (by Bonferroni correction)

Table 4 summarizes the results of neonatal primary outcomes according to ACS therapy exposure. The three groups were similar in sex, birth weight, and incidence of small-for-gestational age or infants with 1-minute Apgar score less than 4 or 5-minute Apgar score less than 7. Neonatal mortality rate was significantly lower in the partial-course group or complete-course group compared to the non-user group, but complete-course ACS therapy was not superior to partial-course therapy. Reduced risk of neonatal mortality with partial- or complete-course ACS therapy was confirmed by multivariable analysis (Table 5). However, ACS therapy (partial- or complete-course) was not associated with a lower rate of infant mortality.

**Table 4:** Neonatal outcome according to exposure to antenatal corticosteroid therapy.

	Non—user (n = 53)	Partial-course (n = 44)	Complete-course (n = 50)	P-value^a^
Sex (Male)^c^	22 (41.5%)	21 (47.7%)	32 (64.0%)	0.065
Birth weight (g, mean±SD)	544.6±91.7	564.4±84.5	546.3±103.9	0.530
SGA	12 (22.6%)	8 (18.2%)	8 (16.0%)	0.682
1 min Apgar score <4	29 (54.7%)	18 (40.9%)	21 (42.0%)^b^	0.302
5 min Apgar score <7	31 (58.5%)	20 (45.5%)	21 (42.0%)	0.211
NICU admission	53 (100%)	44 (100%)	50 (100%)	-
Duration of NICU stay (days, median [range])^c^^,^^d^	25 [1–321]	108 [1–228]	118 [1–380]	0.045
Ventilator treatment	53 (100%)	44 (100%)	50 (100%)	-
Duration of assisted ventilation (days, median [range])^e^	25 [1–194]	42.5 [1–141]	43 [1–270]	0.302
Neonatal mortality^c^	28 (52.8%)	12 (27.3%)^b^	14 (28.0%)^b^	0.010
Infant mortality	34 (64.2%)	19 (43.2%)	22 (44.0%)	0.057
Neonatal morbidity^f^				
RDS	53/53 (100%)	44/44 (100%)	50/50 (100%)	-
BPD	25/25 (100%)	32/32 (100%)	35/36 (97.2%)	0.449
IVH (≥grade 3)^c^	23/42 (54.8%)	18/37 (48.6%)	9/44 (20.5%)^b^	0.003
PVL	5/42 (11.9%)	6/36 (16.7%)	8/44 (18.2%)	0.708
PDA	38/40 (95.0%)	32/37 (86.5%)	38/44 (86.4%)	0.358
ROP (≥grade 3)	9/25 (36.0%)	10/28 (35.7%)	13/33 (39.4%)	0.947
NEC (≥stage 2)	10/35 (28.6%)	9/36 (25.0%)	11/45 (24.4%)	0.907
Early sepsis	33/43 (76.7%)	33/41 (80.5%)	27/46 (58.7%)	0.052
Late sepsis	28/38 (73.7%)	22/36 (61.1%)	23/42 (54.8%)	0.208
Neurodevelopmental outcome^g^				
NDI	15/16 (93.8%)	17/21 (81.0%)	24/25 (96.0%)	0.197
Developmental delay by BSID or DDST	14/17 (82.4%)	17/22 (77.3%)	24/25 (96.0%)	0.162
Cerebral palsy	9/18 (50.0%)	15/24 (62.5%)	14/27 (51.9%)	0.659
Cerebral palsy ≥ level III	1/18 (5.6%)	3/24 (12.5%)	0/26 (0%)	0.171

ACS; antenatal corticosteroid, SD; standard deviation, SGA; small-for-gestational age, NICU; neonatal intensive care unit, RDS; respiratory distress syndrome, BPD; bronchopulmonary dysplasia, IVH, intraventricular hemorrhage, PVL; periventricular leukomalacia, PDA; patent ductus arteriosus, ROP; retinopathy of prematurity, NEC; necrotizing enterocolitis, NDI; neurodevelopmental impairment, BSID; Bayley scales of infant and toddler development, DDST; Denver Developmental Screening test

^a^ intergroup difference by analysis of variance or Kruskal Wallis test for continuous variables, and Chi-square test for categorical variables

^b^ significantly different compared to the non-user group (by Bonferroni correction)

^c^ significant trend by the Jonckheere-Terpstra test for continuous variables and linear by linear association for categorical variables

^d^ analyzed with infants admitted to the NICU only

^e^ analyzed with infants treated with assisted ventilation only

^f^ denominators are the numbers of cases who survived until the diagnosis of each morbidity was possible

^g^ denominators are the numbers of cases who survived until the BSID or neurological examination was possible

**Table 5:** Adjusted odds ratio and 95% confidence interval (CI) of neonatal mortality and intraventricular hemorrhage by exposure to ACS therapy.

Outcomes / aOR^a^	Non-user	Partial-course	Complete-course
**Neonatal mortality**	1.000 (Reference)	0.375 (0.141–0.996)	0.173 (0.052–0.574)
**IVH**	1.000 (Reference)	0.772 (0.307–1.943)	0.191 (0.071–0.516)^c^
**Neonatal mortality or IVH**^b^	1.000 (Reference)	0.708 (0.280–1.789)	0.235 (0.096–0.575)^c^

IVH; intraventricular hemorrhage ≥ grade 3

^a^ adjusted odds ratios (aOR) and 95% confidence intervals estimated with non-ACS users as reference. Multiple logistic regression adjusted for sex, birth weight, mode of delivery, multiple births, use of antenatal tocolytics, year of birth, admission-to-delivery interval, and gestational age at delivery

^b^ composite outcome of neonatal mortality and IVH

^c^ significantly lower aOR compared to the partial-course group

The incidence of RDS, BPD, PVL, NEC stage ≥2, ROP grade ≥3, and neonatal sepsis (early or late) were not significantly different among the three groups. However, IVH grade ≥3 was more commonly diagnosed in the non-user group and partial-course group than the complete-course group. We calculated the risk of composite outcome of neonatal mortality and IVH grade ≥3, because some infants with severe IVH can die before brain assessment. Complete-course ACS therapy, but not partial-course ACS therapy, was associated with decreased risk of severe IVH and its composite outcome with neonatal mortality (Table 5). Long-term neurodevelopmental outcomes (any CP, level III or higher grade CP, developmental delay by BSID score or DDST, and NDI) were not significantly different among the three groups. However, significant positive correlations were identified between severe IVH and any type of CP (Pearson’s r = 0.268, P = 0.026) as well as any type of CP and NDI (Pearson’s r = 0.275, P = 0.032).

## Discussion

In this study, we investigated the effect of partial- or complete-course ACS therapy on neonatal outcomes of infants born prior to 24 weeks of gestation. Regardless of treatment schedule completion, ACS therapy was associated with reduced neonatal mortality. Completion of ACS administration has advantages over partial therapy in reducing severe IVH, but did not improve long-term neurological outcomes.

Over the last few decades, the limit of fetal viability has been decreasing due to great advances in obstetrical and neonatal intensive care. As a result, the incidence of extreme preterm infants and their survival is increasing, especially infants before 24 weeks of gestation. In this study, the incidence of preterm births prior to 24 weeks of gestation increased nearly 5-fold during the last 16 years, and there was a decreasing tendency of neonatal mortality over time, although it was not statistically significant. This special population has been one of the major issues in perinatal medicine during recent years, and ACS therapy is one of the major topics of investigation in neonatal management strategies.

Our fundamental hypothesis was that ACS therapy can reduce neonatal mortality or morbidities by promoting development of fetal organs including lung and brain before 24 weeks of gestation [5]. At 21–23 weeks of gestation, the canalicular stage of fetal lung development is almost complete [20]. Because alveolar development (the critical developmental step in lung maturation) can be observed at this time, neonates born at 21–23 weeks of gestation are potentially viable. However, there was no beneficial effect of ACS therapy in reducing RDS rate in our study as RDS occurred in all infants born before 24 weeks of gestation. Instead, a significant reduction in neonatal mortality was identified in women who received ACS therapy regardless of completion of the treatment schedule.

In addition, reduction of IVH grade ≥3 was found by completing ACS schedule. The exact mechanism of beneficial effect of ACS on high grade IVH is unclear. Premature cerebral blood vessels might not tolerate the fluctuation in cerebral blood flow due to post-partum hypertension, hypercapnia, or fluctuation of carbon dioxide partial pressure [21,22]. In preclinical studies, ACS therapy has been shown to enhance pericyte coverage of cerebral blood vessels and to reduce cerebral blood flow by restricting vasodilation in response to hypercapnia [23,24]. Therefore, maturation of cerebral blood vessels and reduction of cerebral blood flow can be proposed as a clinical mechanism. We identified a protective effect against severe IVH confined to the complete-ACS therapy group, which could be due to dose-dependent cerebral vessel maturation from the corticosteroids. Because high-grade IVH is associated with neonatal death and developmental impairments or mortality in infants, prompt administration of ACS therapy is recommended with the goal of completion before birth.

Despite reduction in severe IVH, neurodevelopmental outcomes were not improved by ACS therapy. This might be due to the small sample size because correlation analysis identified a significant positive correlation between severe IVH and any type of CP as well as any type of CP and NDI. The long-term neurodevelopmental effects of ACS remains unclear; however, recent clinical trials and meta-analysis results generally support the role of ACS therapy in extreme preterm birth. Recent meta-analysis revealed that partial- or complete-course ACS therapy improved neonatal survival (relative risk (RR), 1.19; 95% CI, 1.06–1.33) and reduced the risk of cerebral palsy or disabilities [25]. A lower incidence of IVH or PVL has also been described by several randomized controlled trials. Chawla et al. [26] reported a protective effect of ACS in preterm births of 22 to 27 weeks of gestation. It reduced the rate of IVH or PVL by 50%, the rate of neurodevelopmental impairment including cerebral palsy, cognitive impairment, blindness, or deafness by 30%, and the mortality rate in 18–22 months after birth by 44%. Finally, a large multicenter study observed reduction in the rate of hospital death by 41%, rate of IVH grade ≥3 by 42%, and rate of NEC grade ≥2 in births before 29 weeks of gestation by 38% [27]. Our study is different from these studies in that our population was confined to preterm births before 24 weeks of gestation. In addition, our study included the details of ACS exposure and comparison of outcomes in relation to no, partial-, and complete-course ACS therapy. A large-scale retrospective study in a Japanese patient population identified that ACS administration reduced neonatal death in extremely preterm infants born at 22 to 23 weeks of gestation (adjusted hazard ratio, 0.72; 95% CI, 0.53–0.97), but had no beneficial effect on IVH [28]. We also found that the risk of severe IVH was not significantly different when we simply compared non-users and ACS users, but the beneficial effect of ACS was identified by comparing partial- and complete-ACS users. Therefore, subjects with partial- and complete-course therapy can be considered different clinical groups, and detailed comparison of outcomes regarding ACS dosage is required. We also considered the mediation effect of reduction in IVH with administration of ACS on neonatal death. However, the neurodevelopmental outcomes of surviving infants born before 24 weeks of gestation at 18–22 months in our study were poor; greater than 90% had neurodevelopmental impairment, and more than half had cerebral palsy. A longer-term neurodevelopmental outcome (more than 18–22 months) may be needed for more comprehensive understanding of the benefit and risk of ACS therapy prior to 24 weeks of gestation. However, this was not investigated in our study, because longer-term follow up data were not sufficient for the analysis.

Another important issue is that the use of ACS before 24 weeks of gestation could increase potential exposure to multiple courses of ACS. There are several concerns that multiple repeated courses of ACS may have negative effect on fetal neurological development [29,30]. A recent meta-analysis found short-term benefits of less respiratory distress syndrome and fewer serious infant outcomes, but no significant differences in long-term outcome of infants exposed to single or multiple courses of ACS therapy [31]. The current guidelines recommend that a single rescue therapy should be considered when prior course of ACS was given more than 2 weeks previously [32]. However, no studies or guidelines specifically addressed the issue of repeated or multiple course of ACS therapy in women who received an initial ACS therapy before 24 weeks. In our study, statistical analysis on this issue was not possible because there were only two women who received repeated course of ACS.

The limitation of this study is its retrospective nature, making it vulnerable to information bias. Patient distribution was unequal among the three groups, although baseline characteristics were not significantly different. In addition, the sample size was not large enough to compare the effects of partial-, and complete-course ACS therapy in infants born at 21, 22, or 23 weeks of gestation, even though we recruited subjects over a 16-year study period. To compensate for this limitation, we included gestational age at delivery as a confounding variable in the multivariable analysis, and we found a significant reduction of neonatal mortality with partial- or complete-course ACS therapy and a significant reduction of IVH grade ≥3 by complete-course ACS therapy regardless of gestational age at delivery. However, it is still likely that there are unknown confounding factors that allow a baby to receive a partial- or complete-course of ACS therapy. In addition, longer-term neurodevelopmental outcomes (more than 18–22 months) were not investigated in our study because the number of infants with a longer-term follow up data was not sufficient for analysis. Lastly, the generalizability of our study results is limited because this study was performed in a single center and included only Korean patients.

In conclusion, ACS therapy in preterm births before 24 weeks of gestation is beneficial in reducing neonatal mortality and IVH grade ≥3. ACS therapy in these patients should be encouraged because even incomplete corticosteroid administration can reduce neonatal deaths. However, administration of complete course of ACS therapy is the goal because it reduced the risk of high grade IVH. Our results will be useful for parental counseling and decision-making for neonatal management of these extremely early gestation births, because although survival and short-term neonatal outcomes of these infants can improve with ACS therapy, long-term neurodevelopmental outcomes are still very poor.
